# Multi-Omics Analysis and Machine Learning Prediction Model for Pregnancy Outcomes After Intracytoplasmic Sperm Injection–*in vitro* Fertilization

**DOI:** 10.3389/fpubh.2022.924539

**Published:** 2022-06-30

**Authors:** Fangying Chen, Yixin Chen, Qinyun Mai

**Affiliations:** ^1^Department of Gynecology and Obstetrics, The First Affiliated Hospital of Sun Yat-sen University, Guangzhou, China; ^2^Division of Bioinformatics, Center for Synthetic and Systems Biology, Tsinghua University, Beijing, China; ^3^Department of Obstetrics and Gynecology, The First Affiliated Hospital of Sun Yat-sen University, Guangzhou, China

**Keywords:** intracytoplasmic sperm injection-*in vitro* fertilization, differentially methylated genes, pregnancy, cumulus cells, machine learning model

## Abstract

**Background:**

To explore the methylation profiles in cumulus cells (CCs) of women undergoing intracytoplasmic sperm injection–*in vitro* fertilization (ICSI–IVF) and establish a prediction model of pregnancy outcomes using machine learning approaches.

**Methods:**

Methylation data were retrieved from the Gene Expression Omnibus (GEO) database, and differentially methylated genes (DMGs) were subjected to gene set analyses. Support vector machine (SVM), random forest (RF), and logistic regression (LR) were used to establish the prediction model, and microarray data from GEO was analyzed to identify differentially expressed genes (DEGs) associated with the dichotomous outcomes of clinical pregnancy (pregnant vs. non-pregnant). Kyoto Encyclopedia of Genes and Genomes (KEGG) pathway analysis provided multi-dimensional validation for selected DMGs.

**Results:**

A total of 338 differentially methylated CpG sites associated with 146 unique genes across the genome were identified. Among the identified pathways, the prominent ones were involved in the regulation of cell growth and oocyte development (hsa04340, hsa04012, hsa04914, hsa04614, hsa04913, hsa04020, and hsa00510). The area under the curve (AUC) of machine learning classifiers was 0.94 (SVM) vs. 0.88 (RF) vs. 0.97 (LR). 196 DEGs were found in transcriptional microarray. Mapped genes were selected through overlapping enriched pathways in transcriptional profiles and methylated data of CCs, predictive of successful pregnancy.

**Conclusions:**

Methylated profiles of CCs were significantly different between women receiving ICSI-IVF procedures that conceived successfully and those that did not conceive. Machine learning approaches are powerful tools that may provide crucial information for prognostic assessment. Pathway analysis may be another way in multiomics analysis of cumulus cells.

## Background

There is a rising consciousness in the field of reproductive medicine that oocyte quality is a vital limiting factor in female fertility, especially given the steadily rising age of first conception in mothers. Poor oocyte quality results in either polyspermy, arrested embryonic development, or spontaneous abortion. At present, the evaluation of oocytes mainly relies on morphological features that focus little on these cells' quality and competence (1). In addition, despite controlled ovarian stimulation, the current techniques of *in vitro* oocyte maturation (IVM) are still not efficient enough in cases of multiple immature oocytes. Therefore, more insights into non-invasive biomarkers for oocyte maturation and competence are required.

In pre-antral follicles, the oocyte grew accompanied by relatively undifferentiated granulosa cells (GCs). The follicular antrum formed approximately in the end of the oocyte growth phase, when the GCs differentiate into two anatomically and functionally distinct lineages: mural GCs (MGCs) that line the wall of the follicle, playing principally a steroidogenic role, and cumulus cells (CCs), which establish bidirectional functional interaction with oocytes through gap junctions and paracrine factors (2, 3). Herein, CCs provide somatic support for mature oocytes, and together they form a functional unit called cumulus-oophorus complex (COC), responsible for nuclear and cytoplasmic maturation of oocytes.

Studies have shown the age-related genetic and epigenetic alterations in cumulus cells (4). Likewise, there were documents demonstrating epigenetic changes in cumulus cells and its correlation with infertility in endometriosis (5). Therefore, the exploration of epigenetic and transcriptional profiles in cumulus cells and its relevance with infertility is important and necessary.

Although genetic and transcriptional factors reflecting the development and movement of cumulus GCs have been extensively studied (6–9), the recognition of potential epigenetic factors related to oocyte competence remains elusive. Sagvekar et al. (10) used high-throughput next-generation bisulfite sequencing to assess the methylation and transcript expression profiles of differentially methylated genes (DMGs) in 20 women with polycystic ovary syndrome (PCOS) and 20 healthy individuals, finding that the assessed DMGs matched the transcript expression profiles and were closely related to defective follicles in women with PCOS (10). Nevertheless, the role of epigenetic aberrations in CCs in pregnancy outcomes after undergoing intracytoplasmic sperm injection–*in vitro* fertilization (ICSI–IVF) remains unknown. As a result, more research into the relationships between the methylation state of CpG sites and associated gene expression in cumulus cells, as well as their relationship with pregnancy outcomes, is required.

In this study, bioinformatics analyses were initially performed to identify the methylation status and transcriptional profiles of CCs and their relationship with pregnancy outcomes in patients undergoing ICSI, as well as to analyze the predicted functions and pathways of the DMGs. We also applied multiple machine learning approaches that have been used for cancer genomic classification or subtyping to predict pregnancy outcomes based on selected DMGs. Furthermore, multiomics analysis based on Kyoto Encyclopedia of Genes and Genomes (KEGG) pathway analysis of DMGs and differentially expressed genes (DEGs) was performed.

## Methods

**Figure 1:**
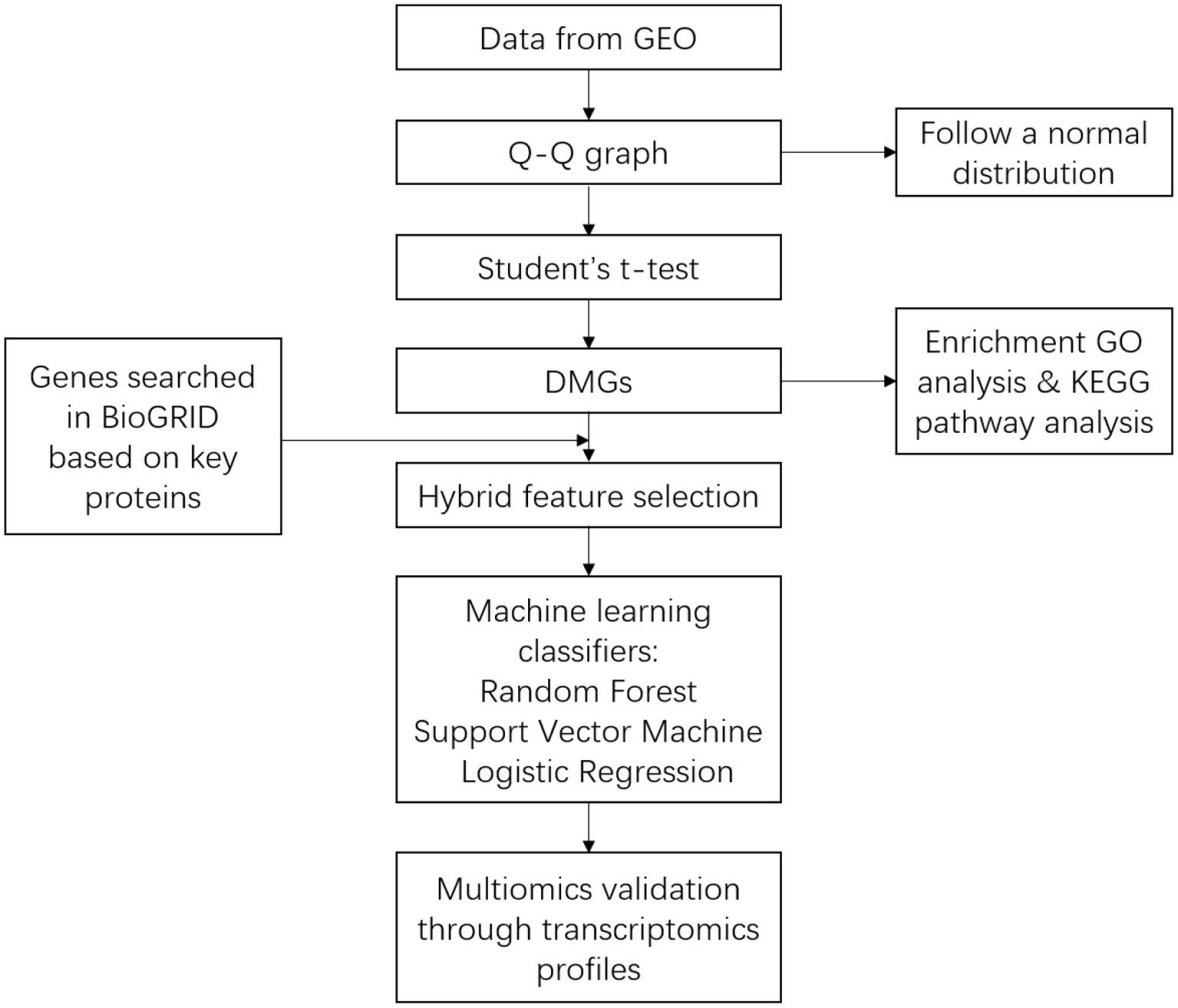
Pipeline showing the approach used for bioinformatic analysis and building the machine learning predictor.

The pipeline used in bioinformatic analysis and building the machine learning model was provided in Figure 1.

### Data Collection

All microarray data were downloaded from the Gene Expression Omnibus (GEO) database (http://www.ncbi.nlm.nih.gov/geo/). The methylation microarray (GSE144664) included 12 IVM-conceived CC samples and 12 IVM-unconceived CC samples based on the HumanMethylation450 BeadChip (GPL13534 platform; Illumina, San Diego, CA, USA). For the same gene corresponding to multiple probes, probes with the largest median absolute deviation were selected as representatives. This study was a second analysis of GEO data; therefore, patient consent was not required.

### Gene Enrichment Analysis

In this study, the Q–Q graph was used to detect whether the sample data followed a normal distribution, and then Student's *t* test was applied to generate a *p*-value. A *p* ≤ 0.05 and a fold change (FC) ≥ 2 were set as the significance thresholds. DMGs related to pregnancy outcomes were analyzed using the Gene Ontology (GO) resource via the ClusterProfiler package (https://bioconductor.org/packages/release/bioc/html/clusterProfiler.html) and KEGG pathway analysis. Only terms with a *p* < 0.05 were considered significant.

### Machine Learning Classifiers

Machine learning methods have been widely applied to many types of pattern-recognition problems. First, principal component analysis (PCA) was applied to the methylated profiles of DMGs, showing a remarkable separating plane between different methylated patterns. Based on the methylation features of CCs, three types of machine learning classifiers, namely support vector machine (SVM), random forest (RF), and logistic regression (LR), were developed for prognosis assessment of pregnancy outcomes following ICSI-IVF procedures.

An SVM is a non-probabilistic supervised learning approach that creates a multidimensional hyperplane to divide the covariate space into two groups that allow for classification. It has been widely used in the analysis of binary results. As originally developed, these models are based on a linear decision surface (hyperplane) that differentiates between two classes of observations and maximizes the distance between the hyperplane and single observations (11). If the classes cannot be separated by a linear surface, a non-linear transformation can be acquired by mapping the data onto a higher-dimensional space (feature space). With the kernel function, this non-linear transformation can be obtained without explicit mapping of the feature space. The SVM classifier we applied was based on a radial basis function kernel. As the kernel parameter is tuned in multiple stages, the SVM classifier can make coarse-to-fine evaluations of the importance of the selected features.

An RF classifier contains a set of decision trees and is based on two modules: bagging and random feature selection. In the bagging step, each tree is trained with a round of bootstrapping from the training data. During the training process, the predictive effect of the RF classifier is tested in out-of-bag samples that were not selected in the bootstrap sample. When a tree is growing, the RF classifier randomly selects a subset of features in each split node. The RF classifier circumvents overfitting and stratifies samples by calculating the complex interactions between variables.

In this study, we applied a hybrid feature-selection method. The key proteins in oocyte–CC paracrine signaling and the sex-hormone-regulation axis were selected and used for searches in Biological General Repository for Interaction Datasets (BioGRID; https://thebiogrid.org/) for matching genes. Subsequently, 32 BioGRID-matched genes and DMGs were selected as the molecular bases for the machine learning classifiers.

Using test data, a trained machine learning model yields a vector of scores (between 0 and 1) that represent negative or positive prediction results. The receiver operating characteristic (ROC) curve was plotted to display the false-positive (FP; 1-specificity) values on the X-axis, and the true-positive (TP; sensitivity) values were plotted on the Y-axis. Thus, the ROC plots display the direct association between the FP and TP rates. The total area under the curve (AUC) for the ROC plots was applied to evaluate the predictive performance of the classifiers.

The predictive classifiers based on 32 DMGs were developed using the Python programming language and the scikit-learning package (Python v3.7; https://www.python.org/). A 50–50% train-test data split was used. In SVM, The RBF kernel (K) was selected since it is a Gaussian function that maps the original feature map to a non-linear space (12), based on data distribution. The number of estimators of RF was optimized as twenty-five in the prediction model according to the efficacy of the RF model. The L2 regularization was applied in LR model and the penalty parameter was set as 1.0. ROC curves were plotted, and AUC values were calculated to describe the predictive efficacy.

### Multi-Omics Analysis Using Transcription Profiles

The transcription file GSE113239 (GPL18451) from the GEO database was used to provide multi-dimensional validation data for the selected DMGs. The prospective clinical trial included 10 women (age: 23–35 years) with idiopathic infertility. Over a period of 14 months, CC gene expression in 10 CC clusters associated with each oocyte was individually evaluated by microarrays using a NimbleGen human gene expression 12 × 135K microarray kit (Roche, Penzberg, Germany). Microarray data from the sequencing of 10 CCs were analyzed to identify DEGs associated with the dichotomous outcomes of clinical pregnancy (pregnant vs. non-pregnant). Data were preprocessed to generate a standard preprocessing structure. Significant genes from these analyses (*p* < 0.05) were selected, and statistical analyses of differential gene expression was performed using the Python programming language and the scikit-learning package (Python v3.7). KEGG analyses were performed using the ClusterProfiler package in R.

## Results

### Result 1 Identification of DMGs

**Figure 2:**
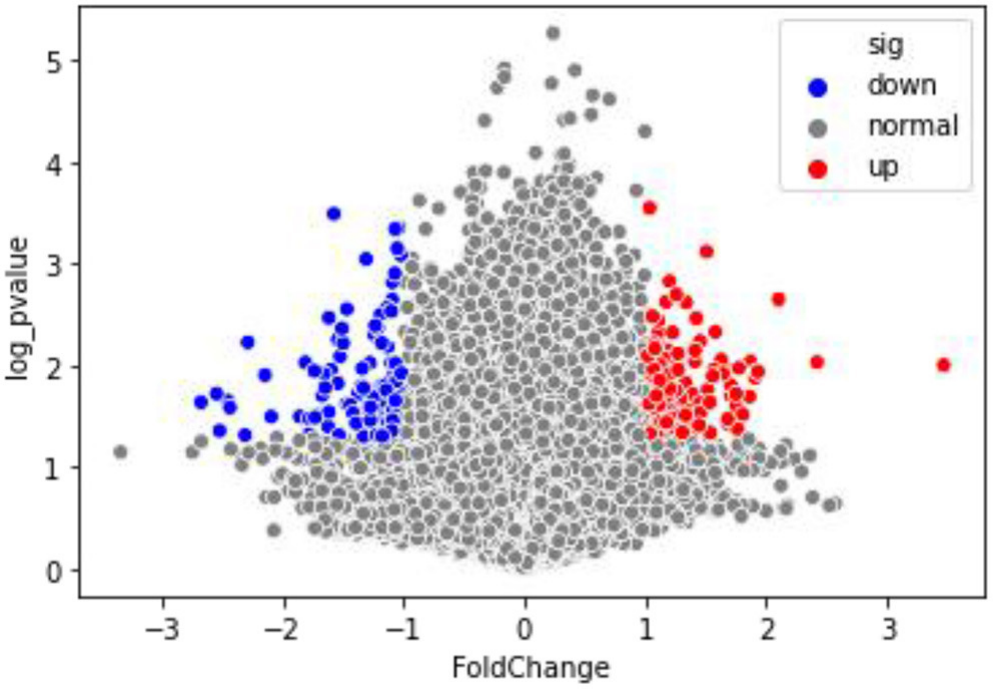
The volcano map of DMGs.

The Q–Q graph showed that the sample data followed a normal distribution (Supplementary Figure 1). Genes above the significance threshold (*p* < 0.05, FC ≥ 2) were established as DMGs. Methylation capture bisulfite sequencing of CCs from women receiving ICSI–IVF identified 338 differentially methylated CpG sites that correlated with 146 unique genes across the genome. Among them, 164 CpG sites were hypomethylated, and 174 CpG sites were hypermethylated. A total of 71 genes matched with hypomethylated CpG sites (Supplementary Table 1), whereas the hypermethylated sites were representative of 78 genes (Supplementary Table 2). Of these DMGs, three (*PQBP1, SEPT6*, and *TIMM17B*) contained both hypermethylated and hypomethylated CpG sites. The volcano map (Figure 2) was visualized for these DMGs.

### Result 2 Enrichment Analysis of DMGs

**Figure 3:**
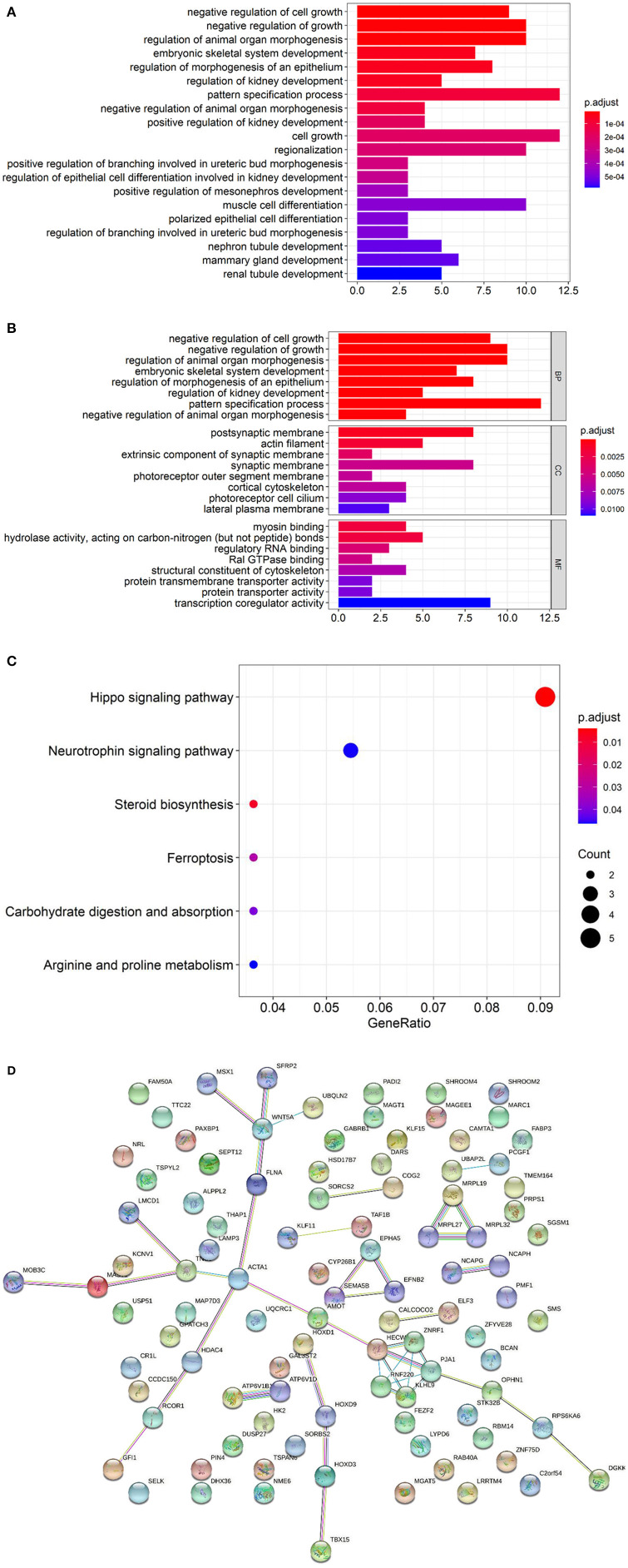
**(A)** GO enrichment analysis of DMGs. The color intensity of the bars represents the number of enriched genes. **(B)** GO enrichment analysis of DMGs divided into BP, CC and MF. BP, Biological Process; CC, Cellular Component; MF, Molecular Function. **(C)** KEGG enrichment analysis of DMGs. **(D)** The functional protein association network of DMGs obtained from the String database.

**Figure 4:**
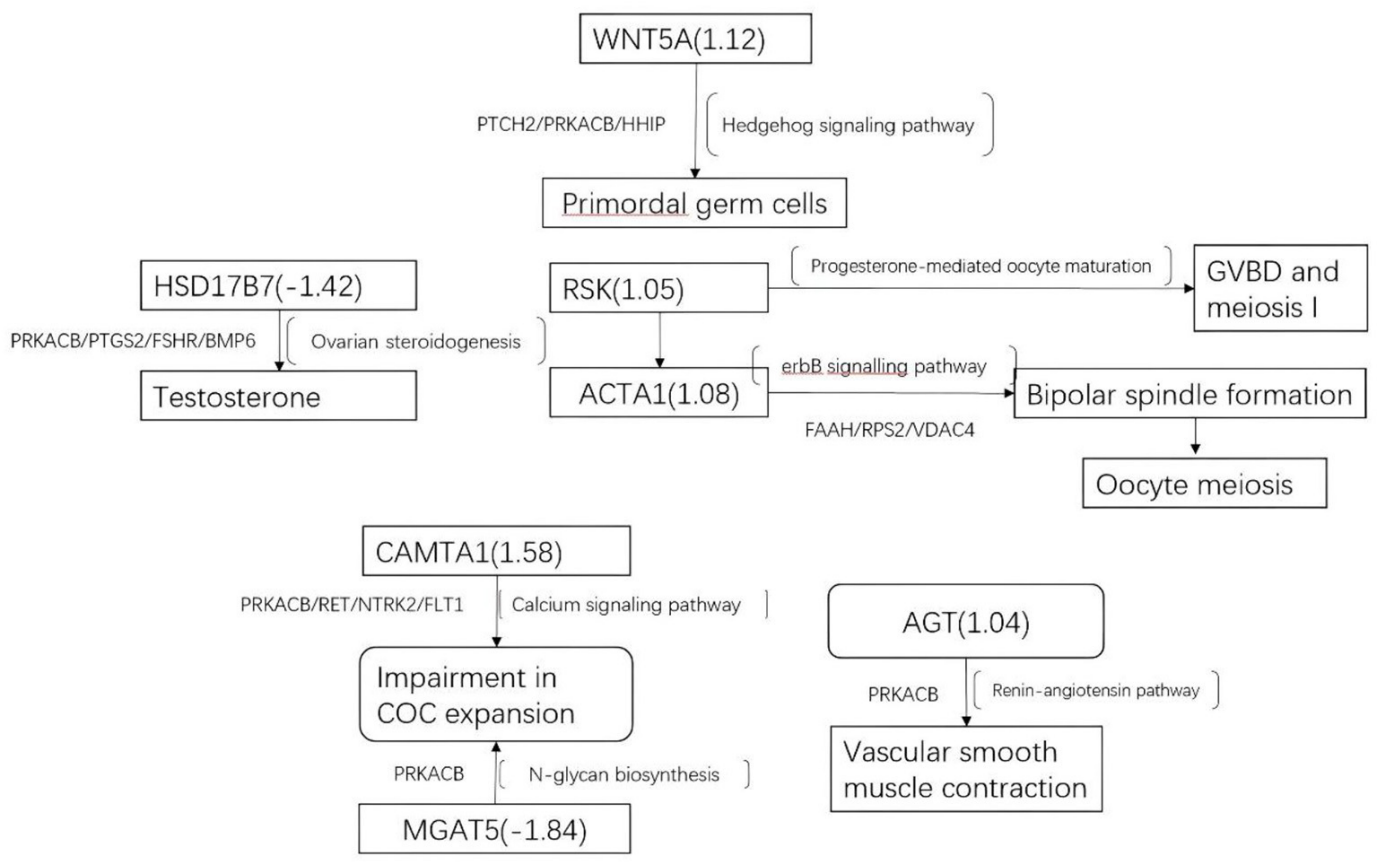
Altered gene methylation and expression and the associated pathways. The figure summarizes some of the processes that could be dysregulated in the follicular compartment, including altered methylation (squares) and expression of genes in cumulus GCs. Differentially methylated genes: *ACTA1, AGT, calmodulin-binding transcription activator 1 (CAMTA1), COC, germinal vesicle breakdown (GVBD), HSD17B7, MGAT5, RSK, and WNT5A. Differentially expressed genes: protein kinase cAMP-activated catalytic subunit beta (PRKACB), prostaglandin-endoperoxide synthase 2 (PTGS2), follicle-stimulating hormone receptor (FSHR), BMP6, patched 2 (PTCH2), Hedgehog-interacting protein (HHIP), integrin subunit beta 8 (ITGB8), integrin subunit alpha L (ITGAL), G protein subunit alpha 13 (GNA13), Rho guanine nucleotide exchange factor 4 (ARHGEF4), ret proto-oncogene (RET), neurotrophic receptor tyrosine kinase 2 (NTRK2), and fms-related receptor tyrosine kinase 1 (FLT1)*.

GO analyses of the hypomethylated, hypermethylated, and combined gene lists revealed that 14 (9.6%), 11 (7.5%), and 13 (8.9%) genes from the three respective groups could not be annotated. DMGs were mainly enriched in negative regulation of growth and organ morphogenesis (Figures 3A,B). For pathway analysis, most involved in Hippo signaling pathway and neurotrophin signaling pathway (Figure 3C). In the Figure 3D, the functional protein association network was obtained from the String database (https://string-db.org). KEGG analysis identified prominent pathways that included Hedgehog signaling (hsa04340), ErbB signaling (hsa04012), progesterone-mediated oocyte maturation (hsa04914), the renin–angiotensin pathway (hsa04614), ovarian steroidogenesis (hsa04913), calcium signaling (hsa04020), and N-glycan biosynthesis (hsa00510) (Figure 4).

### Result 3 Selection of Key Genes and Establishment of Machine Learning Classifiers

**Figure 5:**
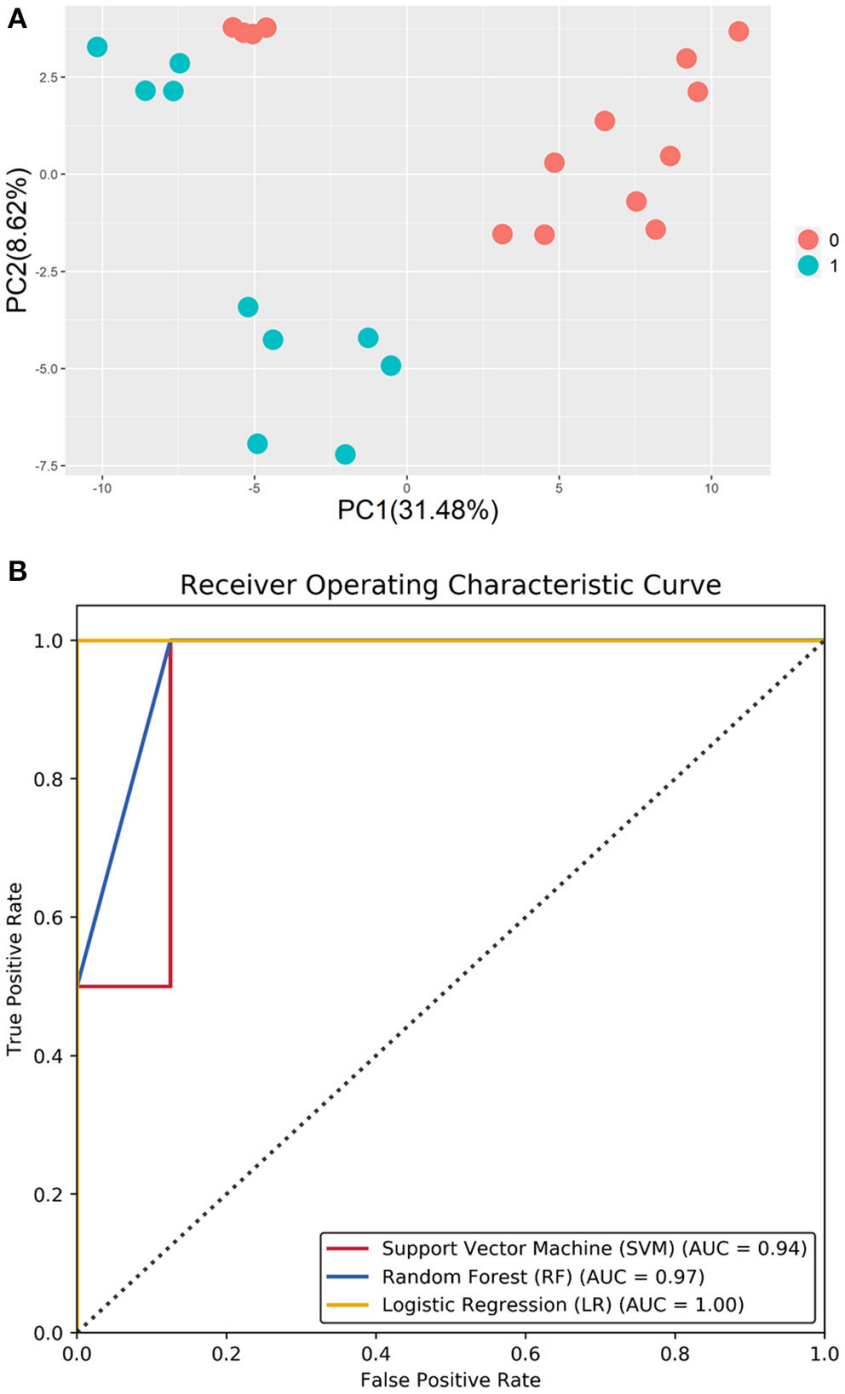
**(A)** PCA of the methylation profiles of DMGs, showing a remarkable separating plane between different methylation patterns. **(B)** Area under the curve (AUC) of the machine learning classifier.

Literature review identified four regulatory proteins secreted by oocytes (BMP6, BMP15, TGFβ, and GDF9) and that act on CCs, with seven maternal upstream regulatory proteins (FSHR, LHCGR, IGF1, ESR1, AR, INHBE, and ACVR1B) were selected as search keywords in BioGRID. The results returned a total of 2,962 related records and 851 non-repetitive interactive genes (Table 1). The 32 intersected genes from among the BioGRID-matched genes and DMGs were then selected as the molecular bases for the machine learning prediction models (Table 2). Figure 5A shows the result of ROC curve analysis, with AUC values for the machine learning classifiers of 0.94 (SVM), 0.88 (RF), and 0.97 (LR) (Figure 5B).

**Table 1:** Interactive genes identified via BioGRID according to regulatory proteins secreted by oocytes and maternal upstream regulatory protein.

**Regulatory genes**	**Aliases**	**Interactive genes**
*BMP6*		8
*BMP15*		5
*TGFB1*	*CED, DPD1, LAP, TGFB, TGF-β*	264
*GDF9*		53
*FSHR*	*FSHRO, LGR1, ODG1*	35
*LHCGR*	*HHG, LCGR, LGR2, LH/CG-R, LH/CGR, LHR, LHRHR, LSH-R, ULG5*	4
*IGF1*	*IGF-I, IGF1A, IGFI*	22
*ESR1*	*ER, ESR, ESRA, ESTRR, Era, NR3A1, RP1-130E4.1*	2155
*AR*	*AIS, DHTR, HUMARA, HYSP1, KD, NR3C4, SBMA, SMAX1, TFM, RP11-383C12.1*	342
*INHBE*	*Inhibin, beta E*	18
*ACVR1B*	*Activin A receptor, type IB*	56

**Table 2:** Overlapping genes between BioGRID and DMGs.

**Genes**	**Log_**2**_ FC**	**Genes**	**Log_**2**_ FC**
*ACTA1*	1.08	*MECP2*	−1.41
*AGT*	1.04	*MGAT5*	−1.84
*BCOR*	1.09	*MORF4L2*	−1.48
*CAMTA1*	1.58	*NCAPH*	−2.69
*CASZ1*	−2.06	*PMF1*	1.80
*CETN2*	1.30	*PQBP1*	−1.03
*CITED1*	−1.02	*RBMS1*	1.24
*CTNNA2*	−1.26	*RPS6KA6*	1.05
*DARS*	1.18	*SAT1*	−1.07
*FLNA*	1.29	*TAF1B*	1.20
*HDAC4*	−1.73	*TDGF1*	−1.71
*HOXD9*	−1.08	*TNS1*	−1.31
*HSD17B7*	−1.42	*UBAP2L*	1.26
*LGALS8*	2.17	*UQCRC1*	−1.00
*MAGED1*	−1.47	*WNT5A*	1.12
*MAGT1*	−1.13	*ZMYM3*	−1.28

### Result 4 Correlation Between Transcriptional and Methylation Files

Additionally, we identified 190 DEGs in the transcription microarray, of which 57 were upregulated and 133 downregulated (Supplementary Table 3). KEGG pathway analysis revealed DEG functions significantly enriched (*p* < 0.05) in signaling pathways that included ovarian steroidogenesis and Hedgehog signaling, which also overlapped with the methylation data (Supplementary Table 4).

**Figure 6:**
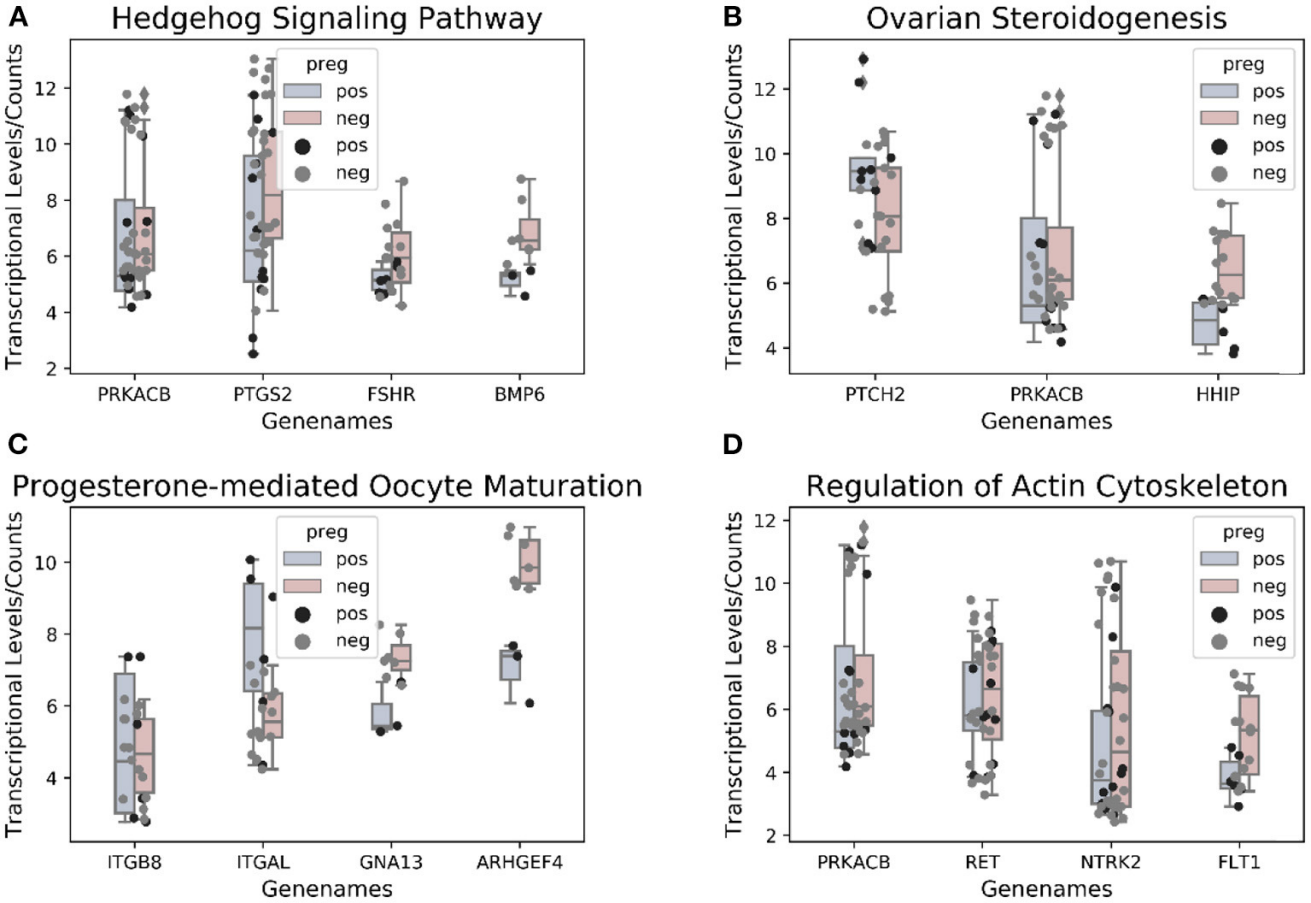
**(A–D)** Genes associated with pathways overlapping between methylation and transcriptional profiles.

The enriched KEGG pathways in the transcription profiles that matched the feature pathway of the methylation profiles are listed in Table 3 along with their correlated genes. These pathways included the Hedgehog signaling pathway, ovarian steroidogenesis, progesterone-mediated oocyte maturation, ErbB signaling pathway/regulation of actin cytoskeleton, the calcium signaling pathway, N-glycan biosynthesis/proteoglycans in cancer, and renin–angiotensin pathway/renin secretion. To illustrate relationships between the transcription and methylation profiles, we mapped the indicated DEGs in the Figure 4. For example, in the N-glycan biosynthesis, Alpha-1,6-mannosylglycoprotein 6-beta-N-acetylglucosaminyltransferase (MGAT5) was negatively methylated in the clinical pregnancy group, while protein kinase cAMP-activated catalytic subunit beta (PRKACB) was negatively expressed (Supplementary Table 3). Figure 6 shows the transcription levels of the mapped DEGs for the positive and negative pregnancy groups. Multi-omics analysis provided biological validation of the selected DMGs used in the machine learning models.

**Table 3:** Overlapping KEGG pathways and pathway-associated genes derived from the GSE113239 (geneID.G11 and SYMBOL.G11) and GSE144664 datasets (geneID.G14 and SYMBOL.G14).

**Description**	**geneID.G14**	**SYMBOL.G14**	**geneID.G11**	**SYMBOL.G11**
cAMP signaling pathway	480	ATP1A4	5567/64399/9568/2492/1387	PRKACB/HHIP/GABBR2/FSHR/CREBBP
Cytokine-cytokine receptor interaction	8784	TNFRSF18	4050/10344/7066/654/9547/56477	LTB/CCL26/THPO/BMP6/CXCL14/CCL28
GABAergic synapse	2752/2560	GLUL/GABRB1	5567/9568/3763/2558	PRKACB/GABBR2/KCNJ6/GABRA5
Leukocyte transendothelial migration	1496	CTNNA2	3683/394/9076/653361	ITGAL/ARHGAP5/CLDN1/NCF1
Morphine addiction	2560	GABRB1	5567/9568/3763/2558	PRKACB/GABBR2/KCNJ6/GABRA5
Ovarian steroidogenesis	51478	HSD17B7	5567/5743/2492/654	PRKACB/PTGS2/FSHR/BMP6
PPAR signaling pathway	2180/2170	ACSL1/FABP3	51129/5105/5346	ANGPTL4/PCK1/PLIN1
Retrograde endocannabinoid signaling	2560/54539	GABRB1/NDUFB11	5567/5743/3763/2558	PRKACB/PTGS2/KCNJ6/GABRA5
Rheumatoid arthritis	525	ATP6V1B1	3683/2321/4050	ITGAL/FLT1/LTB
Transcriptional misregulation in cancer	5081/8842/7849	PAX7/PROM1/PAX8	4211/2321/1050/861/8091	MEIS1/FLT1/CEBPA/RUNX1/HMGA2

## Discussion

Tissue-specific DNA-methylation alterations caused by variations in the physiological environment can lead to significant alterations in gene and protein expression, resulting in disease pathogenesis. Increasing evidence suggests that genomic methylation in CCs plays an important role in oocyte maturation through functional interaction with oocytes. Therefore, we screened the methylation profiles of CCs during IVM cycles. GO and KEGG enrichment analyses of DMGs revealed that genes regulating cell growth, adhesion, differentiation, proliferation, apoptosis, signal transduction, transcription, posttranslational modification, metal and non-metal ion binding, ATP binding, protein binding, and vesicular transport were differentially methylated in the TP group.

### Defective Oocyte Meiosis/Maturation/Ovulation and COC Matrix Expansion

The enrichment of pathways for regulation of focal adhesion elements and cytoskeleton in our analysis indicated deviation of cellular architecture in CCs of women with different pregnancy test results. In this study, calmodulin-binding transcription activator 1 (*CAMTA1*) was significantly hypermethylated in pregnant women; thereby possibly influencing the activity of Ca^2+^/calmodulin kinase (CaMK). Ca^2+^ signaling pathways play a crucial role in the development and maturation of healthy oocytes and are amplified by the activation of the CaMK cascade through successive phosphorylation events (13). In addition, the expression and localization of calcium-sensing receptor (CASR) have been reported in human oocytes and CCs, wherein it mediates an increase in the pronuclear formation rate. The inhibition of CaMK activity could reverse the increase in CASR expression, thus affecting calcium signaling pathway and oocyte development (14).

Ribosomal S6 kinase (RSK) is a pivotal downstream molecule of the mitogen-activated protein kinase (MAPK) cascade, and its activation positively regulates the G2/M transition in mammalian cell lines (15). Several studies have demonstrated its role in progesterone-mediated oocyte meiosis and maturation (16). Madogwe et al. showed that constant ERK1/2 signaling is essential for follicular rupture during ovulation in *RSK3*-knockout mice (17). Hence, hypermethylated *RSK3* may promote clinical pregnancy in infertile women receiving ICSI-IVF through its established positive role in oocyte meiosis, maturation, and ovulation.

### Tumor Necrosis Factor Receptor Superfamily Member 18 and Ovulation

Tumor necrosis factor-alpha (TNF-α) is present in the follicular fluid while its receptors are expressed by GCs. In a group of IVF-ICSI-treated women, TNF-α level in both follicular fluid and serum was found to be higher in good responders than in poor responders (18). Another clinical trial on PCOS showed that lower levels of endogenous TNF-α in GCs contribute to decreased oocyte competence due to a drop in its downstream effector as well as conceded ovulation and GC proliferation in ovarian follicles (10). Thus, lower TNF-α levels in the CCs of women associated with hypermethylation and may affect oocyte competence by hindering COC expansion and hampering ovulation.

### Actin Alpha 1, Skeletal Muscle and Oocyte Meiosis

Several cellular processes are crucial for oocyte meiosis I and II, including nuclear positioning, germinal vesicle breakdown, spindle migration, spindle rotation, chromosome segregation, and polar body extrusion. Several studies, mostly using the mouse oocyte model, have shown that actin filaments are pivotal for these processes, particularly for the formation of bipolar spindles (19–22). In our study, *ACTA1* in CCs was significantly hypermethylated in women with positive pregnancy test results, thus it probably influences oocyte meiosis via the bipolar spindle formation pathway.

### MGAT5 and COC Matrix Expansion

CCs produce extracellular matrix (ECM) molecules, resulting in cumulus expansion that is essential for ovulation and fertilization, and is predictive of oocyte quality (23). As a byproduct of N-glycan biosynthesis, the level of MGAT5 might reflect the status of cumulus expansion that is stabilized by binding N-glycan to matrix proteins and proteoglycans. In addition, a double mutant mouse model revealed that oocyte-specific ablation of core 1 synthase glycoprotein-N-acetylgalactosamine 3-β-galactosyltransferase 1 (C1GALT1) and MGAT1 may reduce Smads 1/5/8 phosphorylation and hyaluronic acid (HA) production by affecting bone morphogenetic protein 15 synthesis or bioactivity, thereby hampering COC matrix expansion (24). Therefore, altered *MGAT5* methylation, as discovered in this study, might contribute to alterations in COC expansion dynamics in women treated with ICSI-IVF.

### Angiotensinogen

Pan et al. found changes in the protein and methylation levels of AGT in the renal tissue of adult IVM mice, suggesting an association between IVM manipulation and epigenetic regulation of the RAS system (25). In our analysis, the association between methylated levels of AGT and pregnancy outcomes was significant, supporting the importance of AGT in the predictive model for pregnancy results.

### Hydroxysteroid 17-Beta Dehydrogenase 7

Hydroxysteroid 17-beta dehydrogenase 7 (HSD17B7) belongs to the hydroxysteroid (17β) dehydrogenase superfamily and acts as a steroidogenic enzyme for the synthesis of arachidonic acid, a precursor of prostaglandins, as well as the conversion of androstenedione to biologically active testosterone in non-testicular tissues and the conversion of estrone to estradiol. The role of HSD17B7 in ovarian function was revealed in HSD17B12+/– ovaries in a female mouse model, in which meiotic spindle formation in immature follicles, polyovular follicles, and oocytes trapped inside the corpus luteum were observed (26). Therefore, hypermethylation of HSD17B7 may result in subfertility through negative effects on meiotic arrest, oogenesis, and ovulation of ovaries.

### WNT

WNT5A is a crucial mediator of the Hedgehog and Wnt signaling pathways that may regulate the formation of primordial germ cells. Akino et al. showed that the transcription levels of *WNT9B/WNT10B/ Frizzled-7/ AXIN2*, which are involved in the Wnt signal/β-catenin pathway, were significantly lower in human dysmature CCs than in control cells (27). Habara et al. revealed that Wnt signaling is pivotal for primordial follicle activation due to regulation of the differentiation of pre-granulosa cells and subsequent oocyte maturation, which is another indicator of how Wnt signaling exerts an effect on oocyte quality and subfertility (28).

### Oxidative Stress

ATPase Na+/K+ transporting subunit alpha 4 (ATP1A4) is involved in generating the plasma membrane electrical potential, resulting in an intracellular pool of functional enzymes, which might be re-expressed during early development in response to physiological needs (29). Upregulated *ATP1A4* expression in oocyte maturation and hydration has been previously reported in marine teleost fish (29). The significant methylation levels of *ATP1A4* between IVM-treated women that conceived and those that did not conceive further indicated the association of *ATP1A4* and fertility.

### Machine Learning Models

KEGG pathway analysis and extensive literature review revealed several candidate genes in CCs that can serve as markers of oocyte quality; however, a reliable method for selecting oocytes with pregnancy potential remains a challenge. In this study, we applied three machine learning approaches, namely SVM, RF, and LR, to predict pregnancy outcomes of ICSI procedures. The model applies a hybrid feature selection method that combines the biological process and filter method to find the suboptimal features for the original input features. In the ROC curve analysis, the areas under the ROC curve were 0.94 (SVM) vs. 0.88 (RF) vs. 0.97 (LR). Together, this study demonstrates that machine learning approaches can provide insights into pregnancy outcomes following ICSI-IVF procedures.

### Comprehensive Analysis Combining Transcriptomics and Methylation Profiles

Previous studies aimed to identify transcription biomarkers indicative of pregnancy outcomes as compared with methylation features. In the omics era, different methods including proteomics, metabolomics, and transcriptomics, have been applied for marker identification (Supplementary Table 5). Although some overlaps between transcription and methylation data could be identified in results obtained from CC global expression, others could not. Iager et al. (30) identified 12 markers as the top DEGs between positive and negative samples after microarray analysis, with these subsequently validated in an independent set of CC samples. Additionally, Feuerstein et al. (31) found that three genes (*EFNB2, RGS2*, and *VCAN*) proposed as biomarkers of pregnancy could not be validated in their CC samples. Xu et al. (32) identified a signature of 30 genes expressed in CC that was predictive of live birth, but the classifier signature could not be applied on external datasets with an accuracy above the confidence level of random chance.

Nevertheless, an increasing number of studies using pathway analysis have reported common affected pathways. In those studies, KEGG pathway analyses identified pathways associated with DEGs and others capable of distinguishing positive samples from negative samples. And more recent studies focused on the validation of pathway-related genes. Artini et al. (33) screened 11 phosphoinositide 3-kinase/AKT pathway-associated genes in human CCs and predictive of clinical pregnancy. Testing of the screened DEGs in a mouse model revealed that these DEGs were significantly downregulated in CCs from oocytes capable of producing a pregnancy as compared with those in CCs associated with a negative outcome (33). In this respect, the present data identified several correlated pathways, including those from the intersection of enriched KEGG analysis using DEGs and DMGs, according to pregnancy outcomes of post-ICSI non-pooled CCs. The transcriptional levels of genes regulating the common affected pathways are shown in Figure 6.

### Limitations

The principle limitation of this study is the small sample size that may not account for women that receive IVM–ICSI treatment and might present various etiologies and disparities in demographics. Additionally, the machine learning models require validation using other datasets with a similar background. Moreover, further experimental validation in human trials or suitable animal models will be necessary to confirm these findings.

## Conclusions

Oocyte quality is a critical limiting factor in female fertility, and current evaluation of oocytes mainly relies on morphological features that focus little on cell quality and competence. Moreover, current techniques for *in vitro* oocyte maturation remain inadequately efficient in cases of multiple immature oocytes, suggesting the need for additional non-invasive biomarkers of oocyte maturation and competence. Previous studies focused on genetic and transcriptional factors reflecting the development and movement of cumulus (4–7), the recognition of potential epigenetic factors related to oocyte competence remains elusive. Our study analyzed methylation data in cumulus cells from women undergoing intracytoplasmic sperm injection–*in vitro* fertilization and further, developed machine learning prediction model based on selected key DMGs. In this study, we performed bioinformatics analyses to identify the methylation status and established a prediction model of pregnancy outcomes using machine learning approaches including SVM, RF and LR based on DMGs. Additionally, independent transcriptional data was applied to illustrate the underlying mechanism of selected DMGs through Kyoto Encyclopedia of Genes and Genomes (KEGG) pathway analysis of differentially expressed genes (DEGs).

## Data Availability Statement

Publicly available datasets were analyzed in this study. This data can be found here: https://www.ncbi.nlm.nih.gov/geo/query/acc.cgi?acc=GSE144664; https://ncbi.nlm.nih.gov/geo/query/acc.cgi?acc=GSE113239.

## Author Contributions

FC analyzed and interpreted the patient data regarding cumulus cells (CCs) of women undergoing ICSI–IVF and was a major contributor in building prediction model and writing the manuscript. YC participated in building the prediction model. QM revised the manuscript. All authors read and approved the final manuscript.

## Funding

This work was supported by Clinical Medical Research of China Medical Sciences—Stem Cell Basic Research Project (Grant No. 19020010780), the National Natural Science Found of China (Grant No. 81270750), and the Natural Science Found of Guangdong China (Grant No. 2019A1515011845). The sponsor was involved in study design, writing of the report, and the decision to submit the article for publication.

## Conflict of Interest

The authors declare that the research was conducted in the absence of any commercial or financial relationships that could be construed as a potential conflict of interest.

## Publisher's Note

All claims expressed in this article are solely those of the authors and do not necessarily represent those of their affiliated organizations, or those of the publisher, the editors and the reviewers. Any product that may be evaluated in this article, or claim that may be made by its manufacturer, is not guaranteed or endorsed by the publisher.

## Supplementary Material

Supplementary Figure 1 Q-Q graph of GSE144664 dataset.

Supplementary Table 1 Genes with hypomethylated CpG sites.

Supplementary Table 2 Genes with hypermethylated CpG sites.

Supplementary Table 3 Differentially expressed genes with *p*-value in GSE113239 dataset.

Supplementary Table 4 Enriched KEGG pathway and annotated genes in GSE113239 dataset.

Supplementary Table 5 The expression markers of non-pooled post-ICSI cumulus cells in various studies.

## Abbreviations

ACTA1: actin-α1, skeletal muscle
AGT: angiotensinogen
ATP14A4: ATPase Na+/K+ transporting subunit α4
AUC: area under the curve
BMP: bone morphogenic protein
CaMK: calcium/calmodulin kinase
CCs: cumulus cells
COC: cumulus–oophorus complex
CASR: calcium-sensing receptor
DEG: differentially expressed gene
DMG: differentially methylated gene
FC: fold change
FP: false positive
GC: granulosa cell
GEO: Gene Expression Omnibus
HSD17B7: hydroxysteroid 17β dehydrogenase 7
ICSI–IVF: intracytoplasmic sperm injection–*in vitro* fertilization
KEGG: Kyoto Encyclopedia of Genes and Genomes
LR: logistic regression
MGAT5: α-1,6-mannosylglycoprotein 6-beta-N-acetylglucosaminyltransferase
MGC: mural granulosa cell
PCA: principal component analysis
PCOS: polycystic ovary syndrome
RF: random forest
ROC: receiver operating characteristic
RSK: ribosomal S6 kinase
SVM: support vector machine
TNF: tumor necrosis factor
TP: true positive
WNT: Wnt family member.
