# Supplementary figures and images for: Multi-Omics Analysis and Machine Learning Prediction Model for Pregnancy Outcomes After Intracytoplasmic Sperm Injection–in vitro Fertilization
Source: Front Public Health. 2022 Jun 30;10:924539. doi: 10.3389/fpubh.2022.924539 (PMC9282825; doi:10.3389/fpubh.2022.924539)

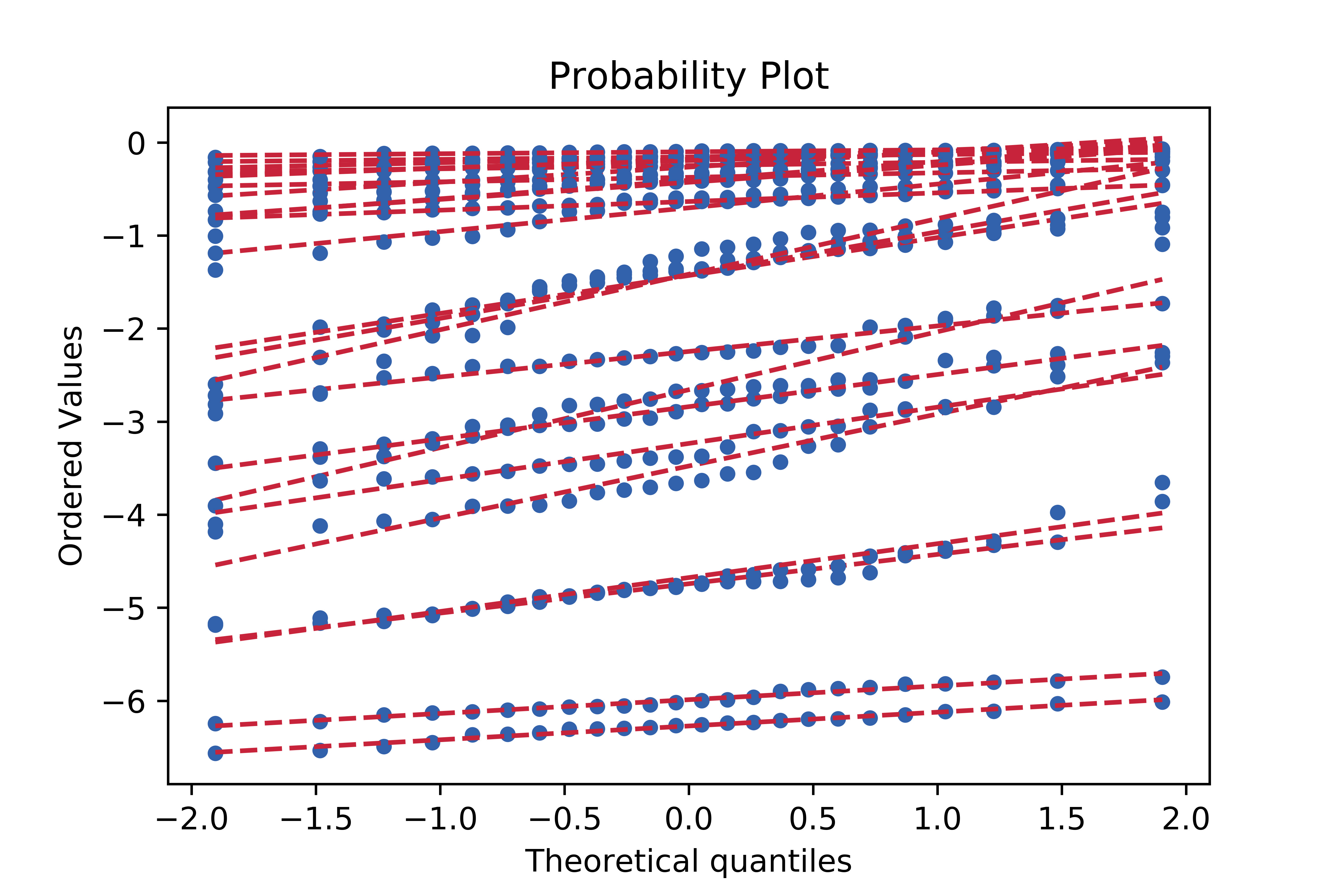

Supplement: Supplementary Figure 1 — Q-Q graph of GSE144664 dataset. [file Image_1.JPEG]
