# Supplementary material for: Multi-Omics Analysis and Machine Learning Prediction Model for Pregnancy Outcomes After Intracytoplasmic Sperm Injection–in vitro Fertilization
Source: Front Public Health. 2022 Jun 30;10:924539. doi: 10.3389/fpubh.2022.924539 (PMC9282825; doi:10.3389/fpubh.2022.924539)
Supplement: Supplementary Table 1 — Genes with hypomethylated CpG sites. [file Table_1.DOCX]

**2.1 Supplementary Table S1. Genes with hypomethylated CpG sites.**

| CpG_hypomethylated | logFC | p value |
| --- | --- | --- |
| CASZ1 | -2.06035 | 0.029402 |
| HSD17B7 | -1.41938 | 0.03249 |
| TNS1 | -1.31406 | 0.039722 |
| ZFYVE28 | -1.02514 | 0.047783 |
| UBQLN2 | -1.10325 | 0.005533 |
| C2ORF54 | -2.26709 | 0.001815 |
| FGF13 | -1.35333 | 0.039727 |
| ZMYM3 | -1.00924 | 0.037042 |
| CCDC150 | -1.02442 | 0.032481 |
| SHROOM2 | -1.28208 | 0.017941 |
| LRRC2; TDGF1 | -1.707 | 0.000642 |
| RAB9B | -1.43614 | 0.029115 |
| HSD17B7 | -1.05553 | 0.007439 |
| UQCRC1 | -1.00478 | 0.024398 |
| HOXD3 | -2.03905 | 0.03321 |
| PRPS1 | -1.49175 | 0.00636 |
| MOBKL2C | -1.34229 | 0.010464 |
| SEPT6 | -1.16785 | 0.004089 |
| ZNF75D | -1.3143 | 0.007473 |
| HDAC4 | -1.73356 | 0.001817 |
| SMS | -1.28881 | 0.009037 |
| FBLN7 | -1.13705 | 0.001945 |
| NME6 | -1.70404 | 0.038883 |
| CLCNKA | -1.19982 | 0.028977 |
| KLHL13 | -1.05235 | 0.047197 |
| LRRTM4 | -1.32749 | 0.006979 |
| TSPYL2 | -1.77493 | 0.001698 |
| SHROOM4 | -1.18101 | 0.03058 |
| C1ORF106 | -1.56048 | 0.000326 |
| MAGT1 | -1.12963 | 0.008634 |
| DUSP27 | -1.30661 | 0.024458 |
| MAGED1 | -1.47193 | 0.009786 |
| LOC375190 | -1.78966 | 0.004673 |
| TMEM164 | -1.10596 | 0.041222 |
| MECP2 | -1.41312 | 0.025222 |
| NR1I2 | -1.7439 | 0.040714 |
| GPATCH3 | -1.05462 | 0.024116 |
| LOC644145 | -1.26843 | 0.009471 |
| MOSC1 | -1.09948 | 0.005617 |
| HOXD9 | -1.08358 | 0.016795 |
| CR1L | -1.07446 | 0.012269 |
| APOBEC4; RGL1 | -2.13109 | 0.001021 |
| SNORA14B; TOMM20 | -1.90229 | 0.045781 |
| STARD7 | -1.5305 | 0.031809 |
| ZMYM3; BCYRN1 | -1.27546 | 0.018949 |
| NCAPH | -2.68821 | 0.004969 |
| AMOT | -1.43619 | 0.010465 |
| ARIH2; C3orf71 | -1.10138 | 0.022372 |
| SLC30A2 | -1.24991 | 0.001631 |
| KLF11 | -1.0926 | 0.030022 |
| TSPAN6 | -1.5392 | 0.026439 |
| GABRB1 | -1.49973 | 0.010861 |
| ALPPL2 | -1.64988 | 0.025548 |
| LYPD6 | -1.15587 | 0.016033 |
| SAT1 | -1.07004 | 0.036994 |
| ZMYM3 | -1.65775 | 0.001237 |
| HK2 | -1.58522 | 0.031499 |
| LAMP3 | -1.49335 | 0.022199 |
| CTNNA2 | -1.26456 | 0.018624 |
| TSPYL2 | -1.95077 | 0.000155 |
| CITED1 | -1.01522 | 0.044658 |
| MRPL19 | -1.18772 | 0.045118 |
| MAP7D3 | -1.11439 | 0.032546 |
| PAX8 | -1.24563 | 0.011237 |
| FAM50A | -1.10817 | 0.034356 |
| MGAT5 | -1.84403 | 0.001625 |
| LOC440905 | -1.31452 | 0.025264 |
| PADI2 | -2.5996 | 0.000458 |
| LMCD1 | -1.56491 | 0.008058 |
| MORF4L2 | -1.47689 | 0.009073 |
| PIN4 | -1.04841 | 0.02014 |
| TIMM17B; PQBP1 | -1.03493 | 0.008663 |
