# Supplementary material for: Multi-Omics Analysis and Machine Learning Prediction Model for Pregnancy Outcomes After Intracytoplasmic Sperm Injection–in vitro Fertilization
Source: Front Public Health. 2022 Jun 30;10:924539. doi: 10.3389/fpubh.2022.924539 (PMC9282825; doi:10.3389/fpubh.2022.924539)
Supplement: Supplementary Table 2 — Genes with hypermethylated CpG sites. [file Table_2.DOCX]

**2.2 Supplementary Table S2. Genes with hypermethylated CpG sites.**

| CpG_hypermethylated | logFC | p value |
| --- | --- | --- |
| ATP1A4 | 2.568979 | 0.002152 |
| ACSL1; SLED1 | 1.103622 | 0.003312 |
| CDA | 1.475132 | 0.013157 |
| AGT | 1.042101 | 0.007381 |
| CNKSR2 | 1.094012 | 0.049352 |
| DMBX1 | 1.328848 | 0.000674 |
| RAB40A | 1.108987 | 0.009405 |
| GAL3ST2 | 1.646754 | 0.02119 |
| EPHA5 | 1.01831 | 0.013571 |
| BCAN | 1.058538 | 0.006358 |
| ATP6V1B1 | 1.040677 | 0.048472 |
| SORBS2 | 1.102183 | 0.041262 |
| MSX1 | 1.187021 | 0.001795 |
| KCND1 | 1.867228 | 0.00286 |
| KCNG3 | 1.424259 | 0.005086 |
| FABP3 | 1.471421 | 0.046009 |
| MAGEE1 | 1.364307 | 0.034324 |
| SFRP2 | 1.002298 | 0.014745 |
| BCOR | 1.093617 | 0.006228 |
| DMD | 1.215718 | 0.002135 |
| STK32B | 1.067596 | 0.009835 |
| TBX15 | 1.008921 | 0.025114 |
| LBX2 | 1.019496 | 0.003899 |
| FLJ42875 | 1.225554 | 0.027788 |
| GLUL | 1.005095 | 0.001604 |
| ACTA1 | 1.077599 | 0.046167 |
| WNT5A | 1.120813 | 0.002321 |
| TIMM8A | 1.92746 | 0.00411 |
| CAMTA1 | 1.57808 | 0.00922 |
| RNF220 | 1.041058 | 0.004163 |
| BCOR | 1.575024 | 0.02647 |
| EGFL6 | 1.131964 | 0.013806 |
| OPHN1 | 1.043014 | 0.017658 |
| COG2 | 1.286023 | 0.024689 |
| SPRR2B | 1.115648 | 0.049018 |
| ARHGAP6 | 1.143059 | 0.049106 |
| TP73 | 1.030749 | 0.00508 |
| ARPM1 | 1.624132 | 0.004288 |
| LGALS8 | 2.167429 | 0.036461 |
| PAX7 | 1.405791 | 0.002107 |
| UBAP2L | 1.262264 | 0.006906 |
| TNFRSF18 | 1.834738 | 0.001776 |
| MOBP | 1.010382 | 0.024863 |
| RPS6KA6 | 1.045324 | 0.034781 |
| NSDHL; CETN2 | 1.302851 | 0.018158 |
| TERC | 1.451307 | 0.007457 |
| SORCS2 | 1.466176 | 0.02686 |
| TTC22 | 1.506197 | 0.046144 |
| TAF1B | 1.197404 | 0.024295 |
| PDE6B | 1.156251 | 0.049934 |
| ZNF673 | 1.437227 | 0.014057 |
| PROM1 | 2.821412 | 0.000049 |
| ATG4A; PSMD10 | 1.018818 | 0.005271 |
| FEZF2 | 1.018344 | 0.030075 |
| DARS | 1.175627 | 0.034596 |
| GFI1 | 1.454187 | 0.009609 |
| SOX2 | 1.0422 | 0.019979 |
| SELK | 1.122539 | 0.010593 |
| USP51 | 1.140969 | 0.005061 |
| HOXD1 | 1.075408 | 0.029326 |
| RBMS1 | 1.237462 | 0.022831 |
| KIF15; KIAA1143 | 1.278637 | 0.00156 |
| SEPT6 | 1.213254 | 0.029048 |
| PQBP1; TIMM17B | 1.328694 | 0.003161 |
| SEMA5B | 1.050475 | 0.015755 |
| CNRIP1 | 1.318419 | 0.001453 |
| DGKK | 1.030732 | 0.031224 |
| NDUFB11; RBM10 | 1.280016 | 0.011929 |
| CYP26B1 | 1.141234 | 0.025769 |
| LOC200726 | 1.197903 | 0.027894 |
| FLNA | 1.289718 | 0.018216 |
| KALRN | 1.086812 | 0.018092 |
| PMF1 | 1.795231 | 0.019467 |
| AJAP1 | 1.322909 | 0.001978 |
| LOC100132215 | 1.098149 | 0.014463 |
