# Supplementary material for: Multi-Omics Analysis and Machine Learning Prediction Model for Pregnancy Outcomes After Intracytoplasmic Sperm Injection–in vitro Fertilization
Source: Front Public Health. 2022 Jun 30;10:924539. doi: 10.3389/fpubh.2022.924539 (PMC9282825; doi:10.3389/fpubh.2022.924539)
Supplement: Supplementary Table 3 — Differentially expressed genes with p-value in GSE113239 dataset. [file Table_3.DOCX]

**2.3 Supplementary Table S3 Differentially Expressed Genes with p-value in GSE113239 dataset**

| Entrez | Genename | Fold | Pvalue |
| --- | --- | --- | --- |
| 4050 | *LTB* | 1.11 | 0.00 |
| 2266 | *FGG* | 1.05 | 0.00 |
| 285025 | *CCDC141* | 1.03 | 0.00 |
| 54621 | *VSIG10* | 0.97 | 0.00 |
| 126393 | *HSPB6* | 0.86 | 0.01 |
| 4653 | *MYOC* | 0.86 | 0.01 |
| 440356 | *CDIPTOSP* | 0.84 | 0.02 |
| 375704 | *ENHO* | 0.82 | 0.02 |
| 221476 | *PI16* | 0.81 | 0.04 |
| 10255 | *HCG9* | 0.78 | 0.00 |
| 154215 | *NKAIN2* | 0.78 | 0.02 |
| 63928 | *CHP2* | 0.78 | 0.03 |
| 3763 | *KCNJ6* | 0.76 | 0.01 |
| 563 | *AZGP1* | 0.75 | 0.03 |
| 4211 | *MEIS1* | 0.71 | 0.02 |
| 50512 | *PODXL2* | 0.71 | 0.05 |
| 3683 | *ITGAL* | 0.70 | 0.03 |
| 8643 | *PTCH2* | 0.69 | 0.02 |
| 440508 | *CLEC4GP1* | 0.68 | 0.01 |
| 6942 | *TCF20* | 0.66 | 0.04 |
| 6891 | *TAP2* | 0.66 | 0.05 |
| 148281 | *SYT6* | 0.65 | 0.00 |
| 51127 | *TRIM17* | 0.65 | 0.02 |
| 51512 | *GTSE1* | 0.64 | 0.02 |
| 9693 | *RAPGEF2* | 0.64 | 0.02 |
| 89765 | *RSPH1* | 0.64 | 0.05 |
| 1850 | *DUSP8* | 0.63 | 0.05 |
| 2015 | *ADGRE1* | 0.63 | 0.04 |
| 9535 | *GMFG* | 0.61 | 0.02 |
| 7832 | *BTG2* | 0.61 | 0.00 |
| 1742 | *DLG4* | 0.61 | 0.03 |
| 130574 | *LYPD6* | 0.60 | 0.03 |
| 10344 | *CCL26* | 0.59 | 0.01 |
| 653361 | *NCF1* | 0.58 | 0.02 |
| 57111 | *RAB25* | 0.58 | 0.00 |
| 79443 | *FYCO1* | 0.57 | 0.03 |
| 197135 | *PATL2* | 0.57 | 0.04 |
| 8000 | *PSCA* | 0.56 | 0.00 |
| 6626 | *SNRPA* | 0.55 | 0.01 |
| 4656 | *MYOG* | 0.55 | 0.00 |
| 654816 | *NCF1B* | 0.55 | 0.02 |
| 266727 | *MDGA1* | 0.54 | 0.01 |
| 53405 | *CLIC5* | 0.53 | 0.02 |
| 7343 | *UBTF* | 0.53 | 0.00 |
| 3696 | *ITGB8* | 0.53 | 0.04 |
| 5099 | *PCDH7* | 0.53 | 0.03 |
| 84182 | *MINDY4* | 0.53 | 0.01 |
| 10590 | *SCGN* | 0.52 | 0.05 |
| 199675 | *MCEMP1* | 0.52 | 0.00 |
| 64753 | *CCDC136* | 0.52 | 0.02 |
| 57817 | *HAMP* | 0.52 | 0.03 |
| 7940 | *LST1* | 0.51 | 0.02 |
| 55063 | *ZCWPW1* | 0.51 | 0.00 |
| 924 | *CD7* | 0.51 | 0.00 |
| 220002 | *CYB561A3* | 0.50 | 0.04 |
| 28996 | *HIPK2* | 0.50 | 0.00 |
| 352954 | *CASTOR3* | 0.50 | 0.02 |
| 23253 | *ANKRD12* | -0.50 | 0.02 |
| 653140 | *FAM228A* | -0.50 | 0.00 |
| 55075 | *UACA* | -0.50 | 0.01 |
| 644903 | *GCC2-AS1* | -0.51 | 0.00 |
| 5346 | *PLIN* | -0.51 | 0.03 |
| 10683 | *DLL3* | -0.51 | 0.03 |
| 22900 | *CARD8* | -0.51 | 0.01 |
| 10672 | *GNA13* | -0.51 | 0.01 |
| 114882 | *OSBPL8* | -0.51 | 0.02 |
| 23350 | *U2SURP* | -0.52 | 0.00 |
| 116987 | *AGAP1* | -0.52 | 0.03 |
| 5444 | *PON1* | -0.52 | 0.01 |
| 55384 | *MEG3* | -0.52 | 0.02 |
| 57531 | *HACE1* | -0.53 | 0.02 |
| 134265 | *AFAP1L1* | -0.53 | 0.03 |
| 154661 | *RUNDC3B* | -0.53 | 0.01 |
| 23682 | *RAB38* | -0.53 | 0.01 |
| 646316 | *TERF1P3* | -0.53 | 0.04 |
| 861 | *RUNX1* | -0.53 | 0.03 |
| 6671 | *SP4* | -0.53 | 0.00 |
| 23413 | *NCS1* | -0.53 | 0.02 |
| 7056 | *THBD* | -0.53 | 0.04 |
| 7066 | *THPO* | -0.54 | 0.01 |
| 10206 | *TRIM13* | -0.54 | 0.04 |
| 64399 | *HHIP* | -0.54 | 0.03 |
| 161742 | *SPRED1* | -0.54 | 0.00 |
| 6866 | *TAC3* | -0.54 | 0.01 |
| 5396 | *PRRX1* | -0.54 | 0.02 |
| 5567 | *PRKACB* | -0.54 | 0.00 |
| 26060 | *APPL1* | -0.54 | 0.00 |
| 84343 | *HPS3* | -0.54 | 0.00 |
| 55554 | *KLK15* | -0.55 | 0.03 |
| 114990 | *VASN* | -0.55 | 0.03 |
| 84153 | *RNASEH2C* | -0.55 | 0.02 |
| 375743 | *PTAR1* | -0.56 | 0.05 |
| 646576 | *HHIP-AS1* | -0.56 | 0.00 |
| 163255 | *ZNF540* | -0.56 | 0.00 |
| 26996 | *GPR160* | -0.56 | 0.00 |
| 134549 | *SHROOM1* | -0.56 | 0.04 |
| 162417 | *NAGS* | -0.56 | 0.05 |
| 23705 | *CADM1* | -0.56 | 0.01 |
| 23576 | *DDAH1* | -0.56 | 0.00 |
| 9201 | *DCLK1* | -0.57 | 0.01 |
| 113230 | *MISP3* | -0.57 | 0.02 |
| 9547 | *CXCL14* | -0.57 | 0.05 |
| 23548 | *TTC33* | -0.57 | 0.00 |
| 375190 | *FAM228B* | -0.57 | 0.00 |
| 51535 | *PPHLN1* | -0.57 | 0.00 |
| 8082 | *SSPN* | -0.57 | 0.00 |
| 285905 | *INTS4P1* | -0.57 | 0.02 |
| 129025 | *ZNF280A* | -0.57 | 0.01 |
| 654 | *BMP6* | -0.57 | 0.02 |
| 5774 | *PTPN3* | -0.58 | 0.02 |
| 5979 | *RET* | -0.58 | 0.01 |
| 6007 | *RHD* | -0.58 | 0.02 |
| 7837 | *PXDN* | -0.58 | 0.03 |
| 79365 | *BHLHE41* | -0.58 | 0.05 |
| 151556 | *GPR155* | -0.59 | 0.05 |
| 1620 | *BRINP1* | -0.59 | 0.01 |
| 11122 | *PTPRT* | -0.59 | 0.04 |
| 54896 | *SLC66A1* | -0.60 | 0.00 |
| 2492 | *FSHR* | -0.60 | 0.02 |
| 8091 | *HMGA2* | -0.60 | 0.01 |
| 84285 | *MGC11102* | -0.60 | 0.00 |
| 440993 | *MIR570HG* | -0.61 | 0.01 |
| 7068 | *THRB* | -0.61 | 0.04 |
| 80036 | *TRPM3* | -0.61 | 0.03 |
| 5105 | *PCK1* | -0.61 | 0.04 |
| 152789 | *JAKMIP1* | -0.61 | 0.03 |
| 394 | *ARHGAP5* | -0.61 | 0.01 |
| 57786 | *RBAK* | -0.61 | 0.03 |
| 38 | *ACAT1* | -0.61 | 0.04 |
| 6785 | *ELOVL4* | -0.62 | 0.03 |
| 23767 | *FLRT3* | -0.62 | 0.03 |
| 160897 | *GPR180* | -0.62 | 0.01 |
| 7552 | *ZNF711* | -0.62 | 0.01 |
| 4915 | *NTRK2* | -0.63 | 0.04 |
| 10417 | *SPON2* | -0.63 | 0.02 |
| 441094 | *NR2F1-AS1* | -0.63 | 0.04 |
| 79698 | *ZMAT4* | -0.63 | 0.00 |
| 80313 | *LRRC27* | -0.63 | 0.02 |
| 112724 | *RDH13* | -0.63 | 0.01 |
| 2256 | *FGF11* | -0.63 | 0.02 |
| 29091 | *STXBP6* | -0.63 | 0.01 |
| 9317 | *PTER* | -0.64 | 0.02 |
| 286046 | *XKR6* | -0.64 | 0.02 |
| 149111 | *CNIH3* | -0.64 | 0.00 |
| 1387 | *CREBBP* | -0.65 | 0.01 |
| 639 | *PRDM1* | -0.65 | 0.00 |
| 162963 | *ZNF610* | -0.65 | 0.00 |
| 9076 | *CLDN1* | -0.66 | 0.01 |
| 65989 | *DLK2* | -0.66 | 0.00 |
| 54852 | *PAQR5* | -0.66 | 0.05 |
| 4330 | *MN1* | -0.66 | 0.02 |
| 64089 | *SNX16* | -0.66 | 0.00 |
| 56106 | *PCDHGA10* | -0.66 | 0.00 |
| 200958 | *MUC20* | -0.67 | 0.02 |
| 5743 | *PTGS2* | -0.67 | 0.03 |
| 25898 | *RCHY1* | -0.67 | 0.01 |
| 6616 | *SNAP25* | -0.68 | 0.02 |
| 284058 | *KANSL1* | -0.69 | 0.01 |
| 92737 | *DNER* | -0.69 | 0.01 |
| 2321 | *FLT1* | -0.70 | 0.00 |
| 79083 | *MLPH* | -0.70 | 0.00 |
| 254122 | *SNX32* | -0.70 | 0.00 |
| 3081 | *HGD* | -0.71 | 0.00 |
| 4504 | *MT3* | -0.71 | 0.03 |
| 79608 | *RIC3* | -0.72 | 0.01 |
| 25803 | *SPDEF* | -0.72 | 0.03 |
| 115273 | *RAB42* | -0.72 | 0.00 |
| 9829 | *DNAJC6* | -0.74 | 0.01 |
| 7036 | *TFR2* | -0.74 | 0.02 |
| 115207 | *KCTD12* | -0.75 | 0.01 |
| 121551 | *BTBD11* | -0.75 | 0.01 |
| 1050 | *CEBPA* | -0.75 | 0.04 |
| 51129 | *ANGPTL4* | -0.76 | 0.00 |
| 57589 | *RIC1* | -0.79 | 0.01 |
| 5730 | *PTGDS* | -0.80 | 0.01 |
| 6781 | *STC1* | -0.82 | 0.02 |
| 115196 | *ZNF554* | -0.82 | 0.05 |
| 2558 | *GABRA5* | -0.83 | 0.01 |
| 89795 | *NAV3* | -0.84 | 0.00 |
| 283755 | *HERC2P3* | -0.85 | 0.01 |
| 55022 | *PID1* | -0.85 | 0.04 |
| 30812 | *SOX8* | -0.88 | 0.00 |
| 56477 | *CCL28* | -0.89 | 0.04 |
| 442523 | *DPY19L2P4* | -0.90 | 0.03 |
| 9568 | *GABBR2* | -0.92 | 0.03 |
| 50649 | *ARHGEF4* | -0.96 | 0.00 |
| 346171 | *ZFP57* | -0.97 | 0.00 |
| 10570 | *DPYSL4* | -0.97 | 0.01 |
| 387978 | *LINC01551* | -1.36 | 0.00 |
