# Supplementary material for: Multi-Omics Analysis and Machine Learning Prediction Model for Pregnancy Outcomes After Intracytoplasmic Sperm Injection–in vitro Fertilization
Source: Front Public Health. 2022 Jun 30;10:924539. doi: 10.3389/fpubh.2022.924539 (PMC9282825; doi:10.3389/fpubh.2022.924539)
Supplement: Supplementary Table 4 — Enriched KEGG pathway and annotated genes in GSE113239 dataset. [file Table_4.DOCX]

**2.4 Supplementary Table S4 Enriched KEGG Pathway and Annotated Genes in GSE113239 dataset**

| KEGG ID | Description | pvalue | geneID |
| --- | --- | --- | --- |
| hsa04913 | Ovarian steroidogenesis | 0.00 | 5567/5743/2492/654 |
| hsa04727 | GABAergic synapse | 0.01 | 5567/9568/3763/2558 |
| hsa05032 | Morphine addiction | 0.01 | 5567/9568/3763/2558 |
| hsa04340 | Hedgehog signaling pathway | 0.01 | 8643/5567/64399 |
| hsa04923 | Regulation of lipolysis in adipocytes | 0.01 | 5567/5743/5346 |
| hsa04670 | Leukocyte transendothelial migration | 0.02 | 3683/394/9076/653361 |
| hsa04062 | Chemokine signaling pathway | 0.03 | 5567/10344/9547/56477/653361 |
| hsa05202 | Transcriptional misregulation in cancer | 0.03 | 4211/2321/1050/861/8091 |
| hsa03320 | PPAR signaling pathway | 0.03 | 51129/5105/5346 |
| hsa04742 | Taste transduction | 0.04 | 5567/9568/2558 |
| hsa04723 | Retrograde endocannabinoid signaling | 0.04 | 5567/5743/3763/2558 |
| hsa04514 | Cell adhesion molecules | 0.04 | 23705/3696/3683/9076 |
| hsa04024 | cAMP signaling pathway | 0.05 | 5567/64399/9568/2492/1387 |
| hsa04060 | Cytokine-cytokine receptor interaction | 0.05 | 4050/10344/7066/654/9547/56477 |
| hsa05323 | Rheumatoid arthritis | 0.05 | 3683/2321/4050 |
| hsa04350 | TGF-beta signaling pathway | 0.05 | 654/57817/1387 |
| hsa04061 | Viral protein interaction with cytokine and cytokine receptor | 0.06 | 10344/9547/56477 |
| hsa04530 | Tight junction | 0.06 | 5567/861/9076/9693 |
| hsa00620 | Pyruvate metabolism | 0.06 | 38/5105 |
| hsa04922 | Glucagon signaling pathway | 0.07 | 5567/5105/1387 |
| hsa05030 | Cocaine addiction | 0.07 | 5567/1742 |
| hsa04726 | Serotonergic synapse | 0.08 | 5567/5743/3763 |
| hsa01212 | Fatty acid metabolism | 0.09 | 38/6785 |
| hsa04919 | Thyroid hormone signaling pathway | 0.09 | 5567/7068/1387 |
| hsa04080 | Neuroactive ligand-receptor interaction | 0.09 | 9568/130574/2492/7068/2558/6866 |
| hsa04330 | Notch signaling pathway | 0.10 | 10683/1387 |
| hsa04611 | Platelet activation | 0.10 | 5567/2266/10672 |
| hsa00590 | Arachidonic acid metabolism | 0.10 | 5743/5730 |
| hsa05217 | Basal cell carcinoma | 0.11 | 8643/64399 |
| hsa04929 | GnRH secretion | 0.11 | 9568/3763 |
| hsa04720 | Long-term potentiation | 0.12 | 5567/1387 |
| hsa05221 | Acute myeloid leukemia | 0.12 | 1050/861 |
| hsa04010 | MAPK signaling pathway | 0.12 | 5567/4915/1850/2321/9693 |
| hsa04915 | Estrogen signaling pathway | 0.12 | 5567/9568/3763 |
| hsa04371 | Apelin signaling pathway | 0.13 | 5567/5346/10672 |
| hsa04810 | Regulation of actin cytoskeleton | 0.13 | 3696/3683/10672/50649 |
| hsa04115 | p53 signaling pathway | 0.14 | 25898/51512 |
| hsa05163 | Human cytomegalovirus infection | 0.14 | 5567/5743/6891/10672 |
| hsa05140 | Leishmaniasis | 0.15 | 5743/653361 |
| hsa01210 | 2-Oxocarboxylic acid metabolism | 0.16 | 162417 |
| hsa04921 | Oxytocin signaling pathway | 0.16 | 5567/5743/3763 |
| hsa04020 | Calcium signaling pathway | 0.16 | 5567/5979/4915/2321 |
| hsa04610 | Complement and coagulation cascades | 0.17 | 2266/7056 |
| hsa04911 | Insulin secretion | 0.18 | 5567/6616 |
| hsa00220 | Arginine biosynthesis | 0.18 | 162417 |
| hsa00900 | Terpenoid backbone biosynthesis | 0.18 | 38 |
| hsa04964 | Proximal tubule bicarbonate reclamation | 0.19 | 5105 |
| hsa04211 | Longevity regulating pathway | 0.19 | 5567/26060 |
| hsa05150 | Staphylococcus aureus infection | 0.21 | 3683/2266 |
| hsa05414 | Dilated cardiomyopathy | 0.21 | 5567/3696 |
| hsa04713 | Circadian entrainment | 0.21 | 5567/3763 |
| hsa00062 | Fatty acid elongation | 0.21 | 6785 |
| hsa01040 | Biosynthesis of unsaturated fatty acids | 0.21 | 6785 |
| hsa01522 | Endocrine resistance | 0.22 | 5567/10683 |
| hsa04640 | Hematopoietic cell lineage | 0.22 | 7066/924 |
| hsa00650 | Butanoate metabolism | 0.22 | 38 |
| hsa04916 | Melanogenesis | 0.23 | 5567/1387 |
| hsa00020 | Citrate cycle (TCA cycle) | 0.23 | 5105 |
| hsa00630 | Glyoxylate and dicarboxylate metabolism | 0.23 | 38 |
| hsa04064 | NF-kappa B signaling pathway | 0.24 | 5743/4050 |
| hsa04613 | Neutrophil extracellular trap formation | 0.24 | 3683/2266/653361 |
| hsa04710 | Circadian rhythm | 0.24 | 79365 |
| hsa04928 | Parathyroid hormone synthesis, secretion and action | 0.24 | 5567/10672 |
| hsa04066 | HIF-1 signaling pathway | 0.25 | 2321/1387 |
| hsa04510 | Focal adhesion | 0.26 | 3696/2321/394 |
| hsa04725 | Cholinergic synapse | 0.27 | 5567/3763 |
| hsa04724 | Glutamatergic synapse | 0.27 | 5567/1742 |
| hsa05168 | Herpes simplex virus 1 infection | 0.27 | 163255/6891/162963/115196/57786/346171 |
| hsa00350 | Tyrosine metabolism | 0.27 | 3081 |
| hsa03030 | DNA replication | 0.27 | 84153 |
| hsa05216 | Thyroid cancer | 0.28 | 5979 |
| hsa04935 | Growth hormone synthesis, secretion and action | 0.29 | 5567/1387 |
| hsa04015 | Rap1 signaling pathway | 0.29 | 3683/2321/9693 |
| hsa05340 | Primary immunodeficiency | 0.29 | 6891 |
| hsa05207 | Chemical carcinogenesis - receptor activation | 0.29 | 5567/10683/54852 |
| hsa05033 | Nicotine addiction | 0.30 | 2558 |
| hsa00380 | Tryptophan metabolism | 0.31 | 38 |
| hsa05166 | Human T-cell leukemia virus 1 infection | 0.31 | 5567/3683/1387 |
| hsa00071 | Fatty acid degradation | 0.32 | 38 |
| hsa04975 | Fat digestion and absorption | 0.32 | 38 |
| hsa04068 | FoxO signaling pathway | 0.32 | 5105/1387 |
| hsa04962 | Vasopressin-regulated water reabsorption | 0.33 | 5567 |
| hsa04728 | Dopaminergic synapse | 0.33 | 5567/3763 |
| hsa02010 | ABC transporters | 0.33 | 6891 |
| hsa04270 | Vascular smooth muscle contraction | 0.33 | 5567/10672 |
| hsa05165 | Human papillomavirus infection | 0.34 | 5567/5743/3696/1387 |
| hsa04014 | Ras signaling pathway | 0.34 | 5567/4915/2321 |
| hsa04910 | Insulin signaling pathway | 0.34 | 5567/5105 |
| hsa00280 | Valine, leucine and isoleucine degradation | 0.35 | 38 |
| hsa05418 | Fluid shear stress and atherosclerosis | 0.35 | 7056/653361 |
| hsa04672 | Intestinal immune network for IgA production | 0.35 | 56477 |
| hsa04979 | Cholesterol metabolism | 0.36 | 51129 |
| hsa05110 | Vibrio cholerae infection | 0.36 | 5567 |
| hsa05144 | Malaria | 0.36 | 3683 |
| hsa03040 | Spliceosome | 0.38 | 6626/23350 |
| hsa04961 | Endocrine and other factor-regulated calcium reabsorption | 0.38 | 5567 |
| hsa04151 | PI3K-Akt signaling pathway | 0.39 | 3696/4915/2321/5105 |
| hsa04144 | Endocytosis | 0.39 | 254122/116987/9829 |
| hsa04145 | Phagosome | 0.39 | 6891/653361 |
| hsa04390 | Hippo signaling pathway | 0.41 | 1742/654 |
| hsa04370 | VEGF signaling pathway | 0.41 | 5743 |
| hsa04730 | Long-term depression | 0.42 | 10672 |
| hsa05416 | Viral myocarditis | 0.42 | 3683 |
| hsa04630 | JAK-STAT signaling pathway | 0.42 | 7066/1387 |
| hsa04213 | Longevity regulating pathway - multiple species | 0.43 | 5567 |
| hsa00310 | Lysine degradation | 0.43 | 38 |
| hsa04310 | Wnt signaling pathway | 0.44 | 5567/1387 |
| hsa04927 | Cortisol synthesis and secretion | 0.44 | 5567 |
| hsa00010 | Glycolysis / Gluconeogenesis | 0.45 | 5105 |
| hsa00830 | Retinol metabolism | 0.46 | 112724 |
| hsa04920 | Adipocytokine signaling pathway | 0.46 | 5105 |
| hsa04924 | Renin secretion | 0.46 | 5567 |
| hsa05031 | Amphetamine addiction | 0.46 | 5567 |
| hsa05204 | Chemical carcinogenesis - DNA adducts | 0.46 | 5743 |
| hsa05211 | Renal cell carcinoma | 0.46 | 1387 |
| hsa05230 | Central carbon metabolism in cancer | 0.47 | 5979 |
| hsa04520 | Adherens junction | 0.47 | 1387 |
| hsa05223 | Non-small cell lung cancer | 0.48 | 5979 |
| hsa01230 | Biosynthesis of amino acids | 0.49 | 162417 |
| hsa04918 | Thyroid hormone synthesis | 0.49 | 5567 |
| hsa04971 | Gastric acid secretion | 0.49 | 5567 |
| hsa05220 | Chronic myeloid leukemia | 0.49 | 861 |
| hsa05034 | Alcoholism | 0.50 | 5567/4915 |
| hsa05412 | Arrhythmogenic right ventricular cardiomyopathy | 0.50 | 3696 |
| hsa04612 | Antigen processing and presentation | 0.50 | 6891 |
| hsa04721 | Synaptic vesicle cycle | 0.50 | 6616 |
| hsa03018 | RNA degradation | 0.51 | 7832 |
| hsa05167 | Kaposi sarcoma-associated herpesvirus infection | 0.52 | 5743/1387 |
| hsa05206 | MicroRNAs in cancer | 0.52 | 5743/8091/1387 |
| hsa05130 | Pathogenic Escherichia coli infection | 0.53 | 10672/9076 |
| hsa05210 | Colorectal cancer | 0.54 | 26060 |
| hsa05169 | Epstein-Barr virus infection | 0.54 | 6891/3683 |
| hsa05203 | Viral carcinogenesis | 0.54 | 5567/1387 |
| hsa04512 | ECM-receptor interaction | 0.55 | 3696 |
| hsa04540 | Gap junction | 0.55 | 5567 |
| hsa04976 | Bile secretion | 0.55 | 5567 |
| hsa05410 | Hypertrophic cardiomyopathy | 0.55 | 3696 |
| hsa04658 | Th1 and Th2 cell differentiation | 0.56 | 10683 |
| hsa05222 | Small cell lung cancer | 0.56 | 5743 |
| hsa04912 | GnRH signaling pathway | 0.57 | 5567 |
| hsa04970 | Salivary secretion | 0.57 | 5567 |
| hsa04657 | IL-17 signaling pathway | 0.57 | 5743 |
| hsa04666 | Fc gamma R-mediated phagocytosis | 0.58 | 653361 |
| hsa05215 | Prostate cancer | 0.58 | 1387 |
| hsa04750 | Inflammatory mediator regulation of TRP channels | 0.58 | 5567 |
| hsa04925 | Aldosterone synthesis and secretion | 0.58 | 5567 |
| hsa04933 | AGE-RAGE signaling pathway in diabetic complications | 0.59 | 7056 |
| hsa04914 | Progesterone-mediated oocyte maturation | 0.60 | 5567 |
| hsa05146 | Amoebiasis | 0.60 | 5567 |
| hsa04625 | C-type lectin receptor signaling pathway | 0.61 | 5743 |
| hsa04714 | Thermogenesis | 0.61 | 5567/5346 |
| hsa04659 | Th17 cell differentiation | 0.62 | 861 |
| hsa04931 | Insulin resistance | 0.62 | 5105 |
| hsa04668 | TNF signaling pathway | 0.63 | 5743 |
| hsa01200 | Carbon metabolism | 0.64 | 38 |
| hsa04071 | Sphingolipid signaling pathway | 0.66 | 10672 |
| hsa04722 | Neurotrophin signaling pathway | 0.66 | 4915 |
| hsa04152 | AMPK signaling pathway | 0.66 | 5105 |
| hsa04110 | Cell cycle | 0.68 | 1387 |
| hsa04380 | Osteoclast differentiation | 0.68 | 653361 |
| hsa04926 | Relaxin signaling pathway | 0.69 | 5567 |
| hsa04114 | Oocyte meiosis | 0.69 | 5567 |
| hsa04650 | Natural killer cell mediated cytotoxicity | 0.69 | 3683 |
| hsa05020 | Prion disease | 0.70 | 5567/653361 |
| hsa05162 | Measles | 0.71 | 25898 |
| hsa04140 | Autophagy - animal | 0.72 | 5567 |
| hsa04120 | Ubiquitin mediated proteolysis | 0.72 | 25898 |
| hsa04550 | Signaling pathways regulating pluripotency of stem cells | 0.72 | 4211 |
| hsa05224 | Breast cancer | 0.73 | 10683 |
| hsa04072 | Phospholipase D signaling pathway | 0.74 | 10672 |
| hsa04261 | Adrenergic signaling in cardiomyocytes | 0.74 | 5567 |
| hsa01240 | Biosynthesis of cofactors | 0.75 | 112724 |
| hsa04932 | Non-alcoholic fatty liver disease | 0.75 | 1050 |
| hsa04934 | Cushing syndrome | 0.75 | 5567 |
| hsa04218 | Cellular senescence | 0.75 | 28996 |
| hsa05160 | Hepatitis C | 0.76 | 9076 |
| hsa05016 | Huntington disease | 0.76 | 1742/1387 |
| hsa05161 | Hepatitis B | 0.77 | 1387 |
| hsa04022 | cGMP-PKG signaling pathway | 0.78 | 10672 |
| hsa05164 | Influenza A | 0.79 | 1387 |
| hsa05152 | Tuberculosis | 0.80 | 1387 |
| hsa04621 | NOD-like receptor signaling pathway | 0.81 | 22900 |
| hsa05415 | Diabetic cardiomyopathy | 0.84 | 653361 |
| hsa05205 | Proteoglycans in cancer | 0.84 | 5567 |
| hsa05170 | Human immunodeficiency virus 1 infection | 0.85 | 6891 |
| hsa05417 | Lipid and atherosclerosis | 0.86 | 653361 |
| hsa05208 | Chemical carcinogenesis - reactive oxygen species | 0.87 | 653361 |
| hsa05171 | Coronavirus disease - COVID-19 | 0.88 | 2266 |
| hsa05132 | Salmonella infection | 0.90 | 79443 |
| hsa05012 | Parkinson disease | 0.91 | 5567 |
| hsa05022 | Pathways of neurodegeneration - multiple diseases | 0.93 | 5743/1742 |
| hsa05010 | Alzheimer disease | 0.97 | 5743 |
| hsa04740 | Olfactory transduction | 0.98 | 5567 |
