# Supplementary material for: Multi-Omics Analysis and Machine Learning Prediction Model for Pregnancy Outcomes After Intracytoplasmic Sperm Injection–in vitro Fertilization
Source: Front Public Health. 2022 Jun 30;10:924539. doi: 10.3389/fpubh.2022.924539 (PMC9282825; doi:10.3389/fpubh.2022.924539)
Supplement: Supplementary Table 5 — The expression markers of non-pooled post-ICSI cumulus cells in various studies. [file Table_5.DOCX]

**2.5 Supplementary Table S5 The Expression Markers of Non-pooled Post-ICSI Cumulus Cells in Various Studies**

| Reference | Stimulation protocol | ICSI/IVF | Isolation method | Number of samples | Detection methods | End point | Selected genes |
| --- | --- | --- | --- | --- | --- | --- | --- |
| Wathlet et al.,2011[1] | Long + Short (17+25) | ICSI | Enzymatic: cumulase enzyme | 42 patients: 142 CCs | Quantitative PCR | Oocyte maturity morphology; pregnancy | SDC4 and VCAN |
| Wathlet et al.,2012[2] | Short | ICSI | Enzymatic: cumulase enzyme | 33 patients: 99 CCs | Quantitative PCR | Clinical pregnancy | EFNB2, CAMK1D, STC1/STC2 |
| Amy E lager et al., 2013[3] | Long + Short | ICSI | Mechanical: scalpel | 55 patients: 101 CCs | Microarray + Quantitative PCR | Live birth | 12 genes |
| Burnik Papler et al.,2015[4] | Short | IVF | Mechanical: scalpel | 43 patients:43 CCs | Quantitative PCR | Clinical pregnancy | EFNB2, RGS2 and VCAN |
| Borup et al., 2016[5] | Long | IVF | Mechanical: 18G needle | 60 patients:27 CCs | Microarray | Live birth | 30 genes |
| Artini et al., 2017[6] | Long | IVF/ICSI | Mechanical: scalpel | 16 donors: 220CCs | Quantitative PCR | Pregnancy outcome | 11 genes |
| J Ekart et al., 2013[7] | Long | ICSI | Mechanical: needle+denudation pipette | 25 patients: 270 CCs | Quantitative PCR | blastocyst formation and live birth | HAS2, FSHR, VCAN and PR |
| Braga et al., 2016[8] | Short | ICSI | Mechanical: 18G needle | 20 patients: 40CCs | LC-MS/MS | Clinical pregnancy | 35 proteins |
| Green et al., 2018[9] | Short | IVF | Mechanical: striping | 17 patients: 34 CCs | RNAseq | Live birth | None |
| Demiray et al., 2019[10] | Long | ICSI | Mechanical: sterile pipette | 10 patients: 10CCs | Microarray | Clinical pregnancy, Live birth | genes related to proteoglycans in the cancer pathway, apoptosis pathways, tumor necrosis factor (TNF) and MAPK pathways, retrograde endocannabinoid signaling, and the transcription factor nuclear factor-κB. |

1. Wathlet S, Adriaenssens T, Segers I, et al. Cumulus cell gene expression predicts better cleavage-stage embryo or blastocyst development and pregnancy for ICSI patients*.* Hum Reprod. 2011;26(5):1035-51.

2. Wathlet S, Adriaenssens T, Segers I, et al. New candidate genes to predict pregnancy outcome in single embryo transfer cycles when using cumulus cell gene expression. Fertility and Sterility. 2012;98(2):432-439.e4.

3. Iager AE, Kocabas AM, Otu HH, et al. Identification of a novel gene set in human cumulus cells predictive of an oocyte's pregnancy potential*.* Fertility and Sterility. 2013;99(3):745-752.e6.

4. Burnik Papler T, Vrtačnik Bokal E, Maver A, et al. Specific gene expression differences in cumulus cells as potential biomarkers of pregnancy*.* Reprod Biomed Online. 2015;30(4):426-33.

5. Borup R, Thuesen LL, Andersen CY, et al. Competence Classification of Cumulus and Granulosa Cell Transcriptome in Embryos Matched by Morphology and Female Age*.* PLoS One. 2016;11(4):e0153562.

6. Artini PG, Tatone C, Sperduti S, et al. Cumulus cells surrounding oocytes with high developmental competence exhibit down-regulation of phosphoinositol 1,3 kinase/protein kinase B (PI3K/AKT) signalling genes involved in proliferation and survival*.* Hum Reprod. 2017;32(12):2474-2484.

7. Ekart J, McNatty K, Hutton J, et al. Ranking and selection of MII oocytes in human ICSI cycles using gene expression levels from associated cumulus cells*.* Hum Reprod. 2013;28(11):2930-42.

8. Braga DP, Setti AS, Lo Turco EG, et al. Protein expression in human cumulus cells as an indicator of blastocyst formation and pregnancy success*.* J Assist Reprod Genet. 2016;33(12):1571-1583.

9. Green KA, Franasiak JM, Werner MD, et al. Cumulus cell transcriptome profiling is not predictive of live birth after in vitro fertilization: a paired analysis of euploid sibling blastocysts*.* Fertil Steril. 2018;109(3):460-466.e2.

10. Demiray SB, Goker ENT, Tavmergen E, et al. Differential gene expression analysis of human cumulus cells*.* 2019;46(2):76.
